# Influence of Age, Host Plant and Mating Status in Pheromone Production and New Insights on Perception Plasticity in *Tuta Absoluta*

**DOI:** 10.3390/insects10080256

**Published:** 2019-08-20

**Authors:** Aroa Domínguez, Sergio López, Ana Bernabé, Ángel Guerrero, Carmen Quero

**Affiliations:** Department of Biological Chemistry, Institute of Advanced Chemistry of Catalonia (CSIC), Jordi Girona 18. 08034 Barcelona, Spain

**Keywords:** pheromone production, olfaction, tomato leafminer, *Tuta absoluta*, electrophysiology, sensitization, autodetection

## Abstract

The tomato leafminer *Tuta absoluta* Meyrick (Lepidoptera: Gelechiidae) is one of the most important pests of tomato worldwide. However, in spite of its tremendous economic importance, the success of environmentally friendly measures to control the pest is still limited. Study of physiological and behavioral parameters that affect pheromone production has provided useful information for pest management. Our results show no clear difference in pheromone production by females over the period from 2 h before to 2 h after the scotophase. However, pheromone production was clearly dependent on female age, with young females producing the highest amount of each pheromone component 10 days after emergence. In the presence of the host plant (physical contact and olfaction of the plant volatiles), virgin and mated females produced higher amounts of the major component of the pheromone (TDTA) than those in the absence of plant and those devoid of olfaction (antennectomized) but in physical contact with the plant. In electrophysiological experiments, TDTA elicited slightly lower responses on male antennae than the pheromone mixture. When stimulated at certain time intervals after the first exposure to TDTA, male antennae became more sensitive to the stimulus (sensitization effect). For the first time in an insect of the family Gelechiidae, we have found that females are able to detect their own pheromone (autodetection). Altogether, our results may represent a step forward in the knowledge of the chemical communication of this important pest.

## 1. Introduction

The tomato leafminer *Tuta absoluta* Meyrick (Lepidoptera: Gelechiidae) is an oligophagous herbivore considered one of the most devastating pests of tomato (*Solanum lycopersicum* L.) crops [1,2,3]. Since its introduction in Eastern Spain in 2006 from South America [4], it has rapidly spread throughout the Mediterranean basin including many European countries, Africa [5], Asia [6], and the Middle East [5,7]. More recently, it has expanded its range to Central America [8] and is a major threat to Mexico, Canada, USA [9], China [10] and India [11,12]. Immediately after invasion, the pest produces high levels of damage, often up to 80–100% yield losses in tomato crops, on greenhouse and open-field tomato production [13]. In addition, *T. absoluta* can also attack potato crops [14,15,16]. Several integrated control measures against *T. absoluta* are being used, e.g., biological control [1,2], essential oils with insecticidal activity [17,18], or optimized agronomic practices, such as fertilization [19,20]. However, in most cases insecticide applications have been required for an effective pest control [21,22] but resistance development has been widely reported [3,23]. Thus, resistance to abamectin [24,25], spinosad [26,27], pyrethroids (λ cyhalothrin, tau fluvalinate) [28], diamide insecticides (chlorantraniliprole, flubendiamide) [29], and to indoxacarb, a bioactive insecticide that requires metabolic activation inside the target insect to express toxicity [30], have been noticed in Greek, Italian, Israelian, and Brazilian populations. These authors also reported the first findings of resistance to indoxacarb, spinosad, and emamectin benzoate in the European-Asian region [7,31]. In addition, insecticides have displayed sublethal effects on predators [32] and parasitoids [33]. Therefore, development of new environmentally friendly control measures, such as the use of pheromones, have been undertaken.

The sex pheromone of *T. absoluta* was identified as a 90:10 mixture of (*E*3,*Z*8,*Z*11)-tetradecatrien-1-yl acetate (*E*3,*Z*8,*Z*11-14:Ac) and (*E*3,*Z*8)-tetradecadien-1-yl acetate (*E*3,*Z*8-14:Ac) [34]. Although both compounds appeared to be important in wind tunnel experiments [35], field tests demonstrated that the presence of the minor component in the pheromone blend did not increase trap catches [36]. Nevertheless, the synthetic pheromone was highly attractive to conspecific males in the laboratory [35] and in the field [36], and therefore used in monitoring populations and detection programs [37] and mating disruption [38,39]. However, pheromone-based control programs have yielded inconsistent results in open field or greenhouses with low levels of confinement. This failure may be partially attributable to the reproductive biology of the pest [3]. Females are polyandrous (they mate once a day and can remate up to six times along their lifespan [40]) and both sexes are able to remate, which allows them to increase fertility, fecundity, and longevity [41]. On the other hand, although sexual reproduction is the predominant strategy, deuterotokous parthenogenesis, which allows production of males and females from unfertilized eggs, has been observed in virgin females under laboratory conditions [42,43]. To the best of our knowledge, there is no documented evidence of wild populations undergoing parthenogenesis in the field. Modulation of pheromone production by physiological factors (age, mating status, …), photoperiod, amount of pheromone release, etc. has been reported in a number of species [44,45]. For instance, the effect of age on pheromone responses has been noticed on the black cutworm *Agrotis ipsilon* (Hufnagel) (Lepidoptera: Noctuidae) [46], the true armyworm *Pseudaletia unipuncta* (HAW.) (Lepidoptera: Noctuidae) [47], the boll weevil *Anthonomus grandis* Boheman (Coleoptera: Curculionidae) [48] or the plum curculio *Conotrachelus nenuphar* Herbst (Coleoptera: Curculionidae) [49]. The amount of pheromone is also an important factor on the behavioral response of the drugstore beetle *Stegobium paniceum* (L.) (Coleoptera: Anobiidae) [50] and the boll weevil [51], and photoperiod has also been recognized to affect pheromone responses in male gypsy moth *Lymantria dispar* Linnaeus (Lepidoptera: Erebidae) [52] and in the cabbage looper *Trichoplusia ni* (Hübner) (Lepidoptera: Noctuidae) [53], among others. Mating status and time since mating also influence emission of sex pheromone components on females of the rice leaf bug *Trigonotylus caelestialium* (Heteroptera: Miridae) with mated females being less attractive to males than virgin females [54]. In this paper, we present for the first time the effect of age, mating status, and presence/absence of the host plant on the pheromone production by *T. absoluta* females, as well as electrophysiological activity of both pheromone components relative to the binary mixture in virgin and mated males and females. 

## 2. Materials and Methods

### 2.1. Insects and Plants

*T. absoluta* moths were reared on tomato plants (*S. lycopersicum* cv*. Early Pack America 3*) provided by the Unit of Plant Physiology (University of Barcelona) at 25 ± 2 °C in a room with 60 ± 10% humidity and 16:8 L:D cycle. During the rearing, no insecticide treatment was applied to the plants. The initial insects were collected in all stages of development from an infested greenhouse in Mataró (Barcelona, Northeast Spain) on May 2014, and renewed annually from the same site. The colony was maintained until it reached 10–12 generations. Tomato plants (20–30 cm high, 34–48 days after planting) were placed individually into a methacrylate cylinder (33 × 20 cm), and covered with finely meshed cloth for ventilation. Each plant was infested with 30 adult males and females in a 1:1 ratio that were supplied with a sugar aqueous solution. Development of the larvae was checked daily and when plants were heavily attacked they were renewed until pupation (3–4 weeks). For virgin males and females, pupae were collected, separated by sex according to morphological discriminatory characters (the presence of two small tubercles on the 8th abdominal segment of female pupae, and the location of the genital opening in each sex) [55], and placed into glass containers (20 × 20 × 5 cm) until emergence. 

### 2.2. Chemicals 

The sex pheromone components *E*3,*Z*8,*Z*11−14:Ac (TDTA) and *E*3,*Z*8−14:Ac (TDDA) were previously synthesized in our laboratory [56], and their purity (>95%) determined by GC or GC-MS analysis. Dodecyl acetate (12:Ac) (≥98%) was purchased from Sigma-Aldrich (Tres Cantos, Madrid, Spain) and used as received. n-Hexane of analytical purity was obtained from Merck (Darmstadt, Germany). 

### 2.3. Pheromone Extracts 

To determine the age of maximum production of pheromone, we analyzed pheromone gland extracts from females of three different age groups (1–4, 5–10 and 11–15 days old). The time period of maximum production of pheromone was determined by analysis of extracts from 2-day-old virgin females at 2 h intervals from 2 h before to 2 h after the scotophase. To study the effect of the mating status and the presence/absence of the host plant in pheromone production, extracts of virgin and mated 2-day-old females, which had been kept isolated (in absence of plant) or in contact with the whole plant (see 2.1. Insects and Plants) for 48 h, were obtained. Females in the presence of plants were subjected to three different treatments: (i) olfaction (females with antennae) and contact with the plant, (ii) olfaction but no contact with the plant; and (iii) no olfaction (antennectomized females) but contact with the plant. Virgin females were directly introduced in the cylinder (see above) (n = 2 females per plant) after emergence. To obtain mated females, newly emerged one female and two males were placed inside the cylinder, observed when mating occurred (males and females are able to mate few hours and about 20–22 h after emergence, respectively) [57], and then males were removed to avoid any possibility of remating. If no mating was observed during the assay, the corresponding female was discarded for gland extraction. Regarding antennectomized insects, antennae of virgin females were excised shortly after their emergence, whereas those of mated individuals were cut off before their introduction in the plant-containing cylinders. Glands were extracted 2 h after the onset of the scotophase, and for each replicate two glands were excised and extracted with 100 µL of hexane at room temperature for 60 min and kept in a freezer at −80 °C until analysis. A total of 7–13 replicates per type of assay were done. 

### 2.4. Chemical Analysis

Gland extracts were analyzed by GC-MS in splitless mode on a Finnigan Trace 2000 GC system (Thermo Fisher Scientific, Alcobendas, Madrid, Spain) coupled to a Trace MS quadrupole mass spectrometer (Thermo Fisher Sci., Waltham, MA, USA) working in electron impact (EI) mode. The analyses were implemented using an HP-5MS 30 m × 0.25 mm i.d. × 0.25 μm fused silica capillary column (Agilent Technologies, Madrid, Spain). The chromatographic conditions were as follows: injection at 60 °C for 1 min and program of 5 °C/min to 240 °C and 10 °C/min to 280 °C, which was maintained for 10 min more. The mass range was m/z 40–500 with a scan time of 1 s. Identification of pheromone compounds was done by comparison of their mass spectra and retention times with those of the synthetic chemicals, as previously described [35,36]. The extracts were carefully concentrated under a nitrogen stream to a final volume of 1–2 µL, and the entire extract was injected in splitless mode. For quantification of the two pheromone components, 10 µL of a 1 ng/µL solution of 12:Ac in hexane was added as internal standard to each gland extract. A separate calibration curve (5–40 ng) was established for the major component and assumed that the chromatographic response factors for the minor compound TDDA would be highly similar to those for TDTA. 

### 2.5. Electroantennogram (EAG) Assays

The EAG apparatus was from Syntech (Buchenbach, Germany) and the methodology used was based on standardized protocols [58]. Briefly, male and female antennae were excised, cut on both ends, and fixed to both electrodes with conducting gel Spectra 360 (Parker Lab. Inc, Hellendoor, Netherlands). A flow of humidified pure air (700 mL/min) was continuously directed over the antenna through the main branch of a glass tube (7 cm long × 5 mm diameter). Test stimulations were implemented by giving puffs of air (160 mL/min) for 100 ms through a Pasteur pipet using a TC-05 stimulus controller (Syntech). The pipette contained a small piece of Whatman filter paper (2.5 cm diameter) on which the tested compound had been deposited at the appropriate concentrations. The solvent (hexane) was allowed to evaporate before the tests. For EAG activity, 1–4-day-old virgin and mated males and females were subjected to two puffs from 1 ng to 100 µg doses in a 10-fold increase of both components of the pheromone and the natural blend (mixture of the major and the minor compound in 9:1 ratio). The puffs were insufflated over the antenna of 9–11 virgin and mated males and females at 60 s intervals and control puffs (hexane) were intercalated between two consecutive stimuli. The output signals were amplified (100 ×), filtered (DC to 1 kHz) with an IDAC-2 interface (Syntech), digitized on a PC and analysed with EAG 2000 program (Syntech). The net EAG responses were calculated by subtracting the mean response to control (hexane) before and after each stimulus from the mean response to the corresponding chemical. For the sensitization effect, the response of 1–4-day-old virgin and mated males to 1 µg of TDTA was evaluated in 8–9 individuals at 0, 9, 13, 19, 24 and 33 min from the beginning of the experiment. The methodology for this assay followed the steps described above, with two consecutive stimuli of 1 µg of the major compound, and solvent puffs before and after the synthetic material. Between each time interval, the antenna was subjected to a permanent humidified airflow (ca. 650 mL/min) to prevent its gradual decay of sensitivity with time.

### 2.6. Statistical Analysis

Prior to the analysis, data were checked to verify that they followed a normal distribution, and if necessary, they were transformed into ln (x + 0.1). For the pheromone gland composition analysis, data were subjected to a one-way analysis of variance (ANOVA) followed by the Tukey test. When the conditions to apply ANOVA were not complied, the non-parametric Kruskal-Wallis test was implemented followed by Z-Kolmogorov-Smirnov test for pairwise comparisons. Comparison of the EAG response of males and females at different doses was performed by ANOVA followed by Tukey post hoc test. To compare the effect of the physiological state on the response to a particular dose, the Student’s T-test was applied. For the sensitization effect, in which each insect displayed 6 sequential and correlated EAG responses over time, the multilevel Linear Random-Intercept Regression Model (LRIRM) was fitted using restricted maximum likelihood (REML) estimation method, taking into account the variability within- and between-insects [59]. Time, mating status and their first order interaction were the covariates of the model at cluster (i.e., insect) or level-2. Percent increases from the predicted values and their 95% confidence intervals were also calculated. All analyses were conducted using the statistical software Stata 12.0 [60] and tests were two-sided for a significance level α < 0.05.

## 3. Results

### 3.1. Pheromone Composition and Effect of Time into Scotophase and Female Age on Pheromone Production

**Figure 1:**
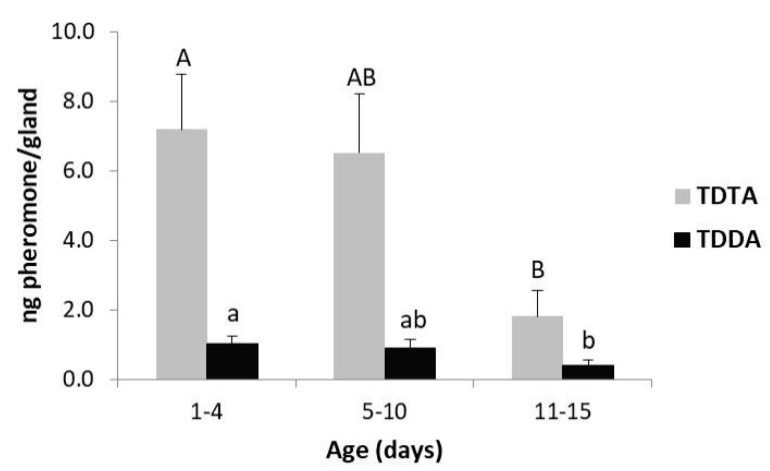
Pheromone contents (mean + SE) of gland extracts of *T. absoluta* females at different ages (N = 8, 4, and 6 for 1–4, 5–10, and 11–15 days of age, respectively). Glands were excised 2 h after the onset of the scotophase. Means followed by the same letter are not significantly different (Tukey test post hoc test, *p* < 0.05).

Analysis of a pool of virgin female gland extracts of *T. absoluta* showed the presence of TDTA as the major, and TDDA as the minor, components respectively of the sex pheromone in a 90:10 ratio (Figure S1, Supplementary Material) [34,35]. There were no significant differences in the production of pheromone over the period 2 h before and after the scotophase (F = 1.2, *df* = 24, *p* = 0.280), with females producing higher amounts of the pheromone at the beginning (8 ± 1.0 ng/gland) and at the end (8.2 ± 1.6 ng/gland) of the period than in the middle (6.5 ± 1.2 to 6.8 ± 0.7 ng/gland) (Figure S2, Supplementary Material). However, the amount of each pheromone component produced was clearly affected by the female age with the amount of TDTA and TDDA being significantly lower in old (>11 days old) than in young females (<4 days old) (F = 4.3, *df* = 17, *p* = 0.033; Figure 1). Maximum pheromone production (7.2 ± 1.6 ng/gland of TDTA, 1.0 ± 0.2 ng/gland of TDDA) was observed during the first 4 days after emergence. The amount of pheromone was steadily similar during 5–10 days before decreasing to 1.8 ± 0.7 ng/gland and 0.4 ± 0.2 ng/gland for the major and minor component, respectively (Figure 1).

### 3.2. Effect of Mating Status and the Presence/Absence of the Host Plant in Pheromone Production

**Figure 2:**
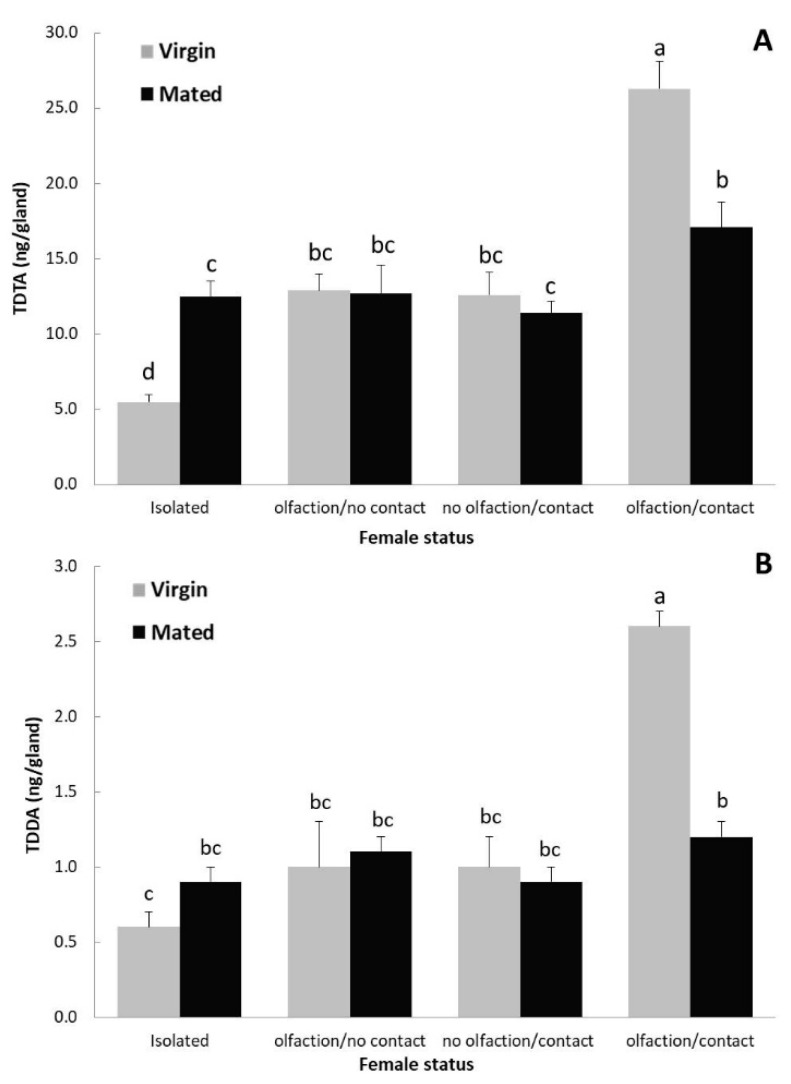
Pheromone components production (mean + SE) (TDTA in **A**; TDDA in **B**) by 2-day old virgin and mated *T. absoluta* females (N = 7–13 extracts) in the absence (isolated) and presence (olfaction/no contact, no olfaction/contact, olfaction/contact) of the host plant. Bars with different letters within the same pheromone component are statistically different (Kolmogorov and Tukey and post hoc test for TDTA and TDDA respectively, *p* < 0.05).

Gland extracts of mated females contained comparable amounts of TDTA in the absence of plants (isolated, 12.5 ± 1.0 ng/gland) to those of individuals that had been in contact only with the leaves (no olfaction) (11.4 ± 0.8 ng/gland) or with no contact (only olfaction) (12.7 ± 1.9 ng/gland) (Figure 2A). In mated females, the largest amount of pheromone was detected when they were totally exposed to the tomato plant (olfaction + contact) (17.1 ± 1.7 ng/gland) although the difference was only partially significant (Figure 2A). No significance was found in the production of TDDA among mated females in all treatments (Figure 2B).

In the absence of the host (isolated insects), virgin females produced significantly lower amounts of either component (5.5 ± 0.5 ng/gland of TDTA and 0.6 ± 0.1 ng/gland of TDDA) than the corresponding mated individuals (12.5 ± 1.0 ng/gland and 0.9 ± 0.1 ng/gland, respectively). In the presence of plant, antennectomized females (no olfaction) in contact with the leaves and intact insects with no contact with the plant produced similar amounts of pheromone regardless of their mating status (12.6–12.9 ng/gland of TDTA and 1.0 ng/gland of TDDA for virgin, and 11.4–12.7 ng/gland of TDTA and 0.9–1.1 ng/gland of TDDA for mated females) (Figure 2A,B). However, virgin females that had been in full contact with the plant produced the highest amount of pheromone in all treatments (26.3 ± 1.8 ng/gland of TDTA and 2.6 ± 0.1 ng/gland of TDDA), significantly higher than mated females (Table S1, Supplementary Material).

### 3.3. Electrophysiological Activity of Both Pheromone Components on Males 

**Figure 3:**
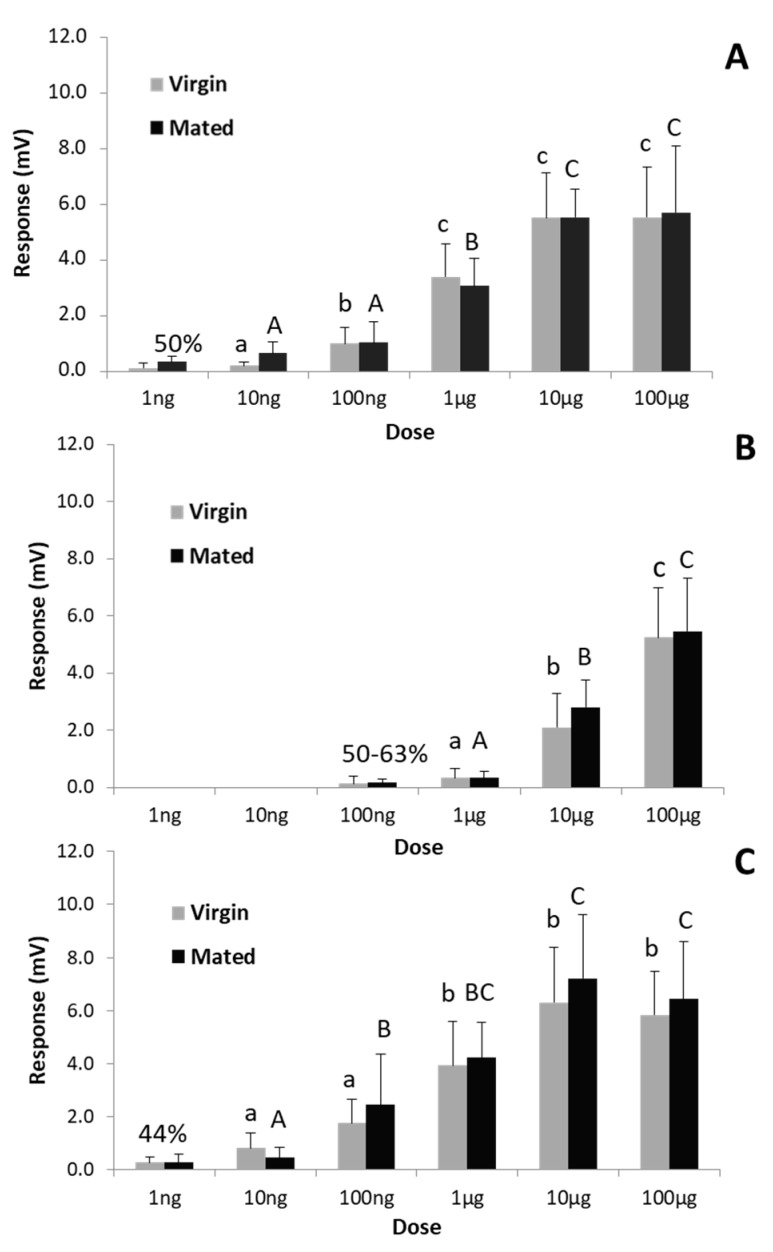
Mean EAG response (+ SE) of antennae of virgin and mated *Tuta absoluta* males (N = 9) to different doses of the pheromone components TDTA (**A**) and TDDA (**B**) and their mixture in 9:1 ratio (**C**). Bars with different letters within the same mating status (in lower case virgin and in capital mated males) are significantly different (Tukey post hoc test, *p* < 0.05). Percentage on bars indicates the number of insects responding to the corresponding dose but the EAG values were not taken into consideration for statistical analysis.

Virgin vs. mated males of 1–4 day-old elicited similar EAG responses to the pheromone components at all doses tested, either when compounds were tested individually or mixed in the natural ratio (9:1) (Figure 3A,C). Responses to the binary mixture were generally similar or slightly higher than those elicited by the major compound alone. In this regard, it should be noted that the amount of the major compound when tested individually was 10% higher than when assayed as the pheromone mixture. This small difference in dose does not affect comparison of the EAG responses to the major component in both cases since the EAG is unable to detect a 10% difference in dose of an specific material. In this respect, EAG doses are usually applied in one order of amplitude differences. For the minor component it was necessary a much higher dose to get the maximum depolarizations (Figure 3B). At the lowest tested dose (1 ng), only 50 and 44% of males displayed a very minor response to TDTA and to the binary mixture, respectively (Figure 3A,C), whereas for TDDA it was necessary at least 100 ng to elicit small but consistent depolarizations on 50–63% of individuals (Figure 3B). The EAG values recorded at doses for which not all males responded were not considered for statistical analysis. 

**Figure 4:**
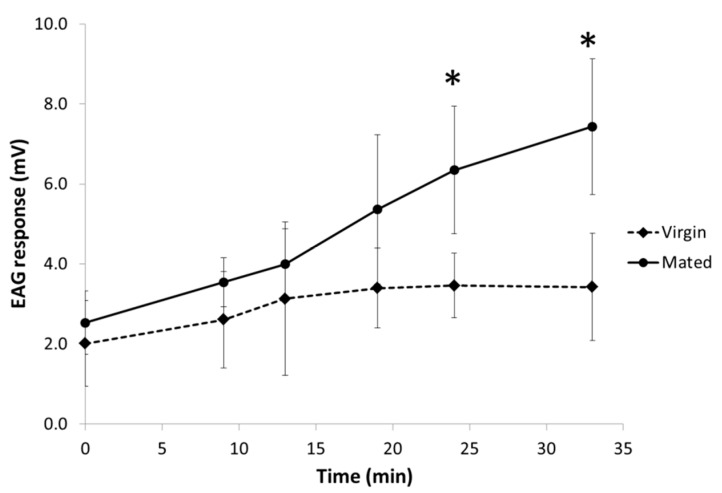
Mean EAG response (mV ± 95% Confidence Interval) of *T. absoluta* male antennae (N = 10–11) when stimulated with 1 µg of TDTA at 9, 13, 19, 24 and 33 min after pre-exposure (T = 0 min). Asterisks denote statistical significance between mating status at the same time of stimulation (LRIRM analysis, *p* < 0.05).

When the male antenna was subjected to puffs of 1 µg of TDTA at different time intervals, the EAG response at T = 0 was lower (1.8 ± 1.1 mV in virgin males and 2.5 ± 0.8 mV in mated males) (Figure 4) than the one elicited at the same dose in the dose-response study shown in Figure 3 (3.4 ± 1.1 mV in virgin and 3.1 ± 1.0 mV in mated males), wherein the insects had been previously exposed to other doses of pheromone. This sensitization effect was confirmed in virgin and mated males when the same male antennae were stimulated at times 9, 13, 19, 24 and 33 min after the first exposure to the pheromone (T = 0 min) (Figure 4). 

In virgin insects, “puffs” of 1 µg of the major component induced a significant increase of response of 47, 70, 91, 88 and 80% at 9, 13, 19, 24 and 33 min, respectively, relative to the response at T = 0 (Table 1). In mated males, the increase of response was also higher (58, 101, 137 and 163% at 13, 19, 24 and 33 min, respectively) than that at T = 0. The responses appeared to be higher in mated than in virgin males, but the differences were significant only after the first 24 min from exposure (Figure 4, Table S2 in Supplementary Material) by LRIRM analysis. 

### 3.4. Electrophysiological Activity of Both Pheromone Components on Females (Pheromone Autodetection)

**Figure 5:**
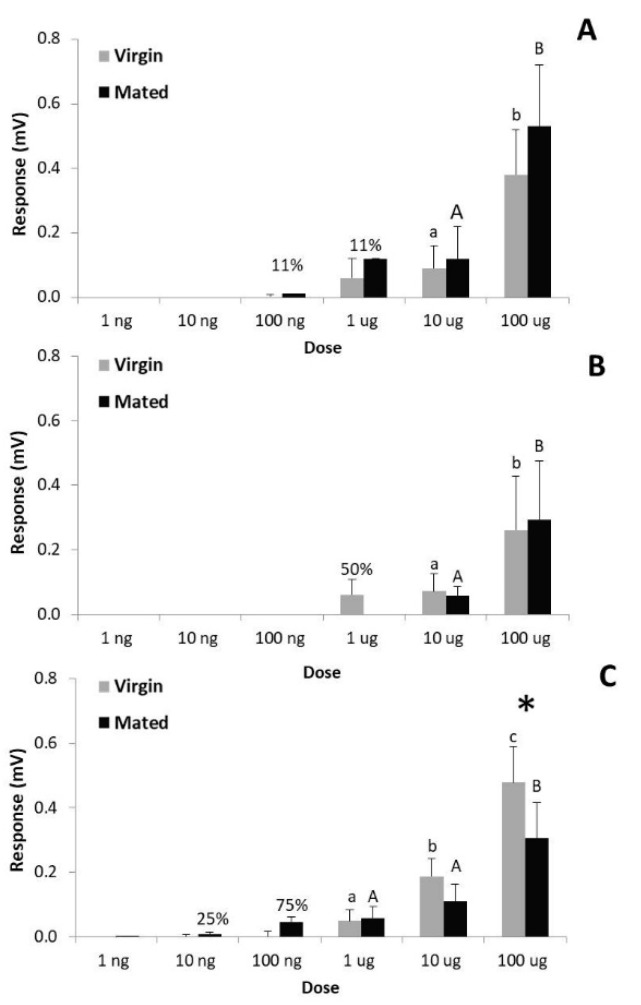
Mean EAG response (+ SE) of female antennae (N = 8–9) of *T. absoluta* to the synthetic pheromone compounds TDTA (**A**) and TDDA (**B**), and to the binary mixture (**C**). Numbers above bars represent percentage of antennae that responded to the specific dose but the corresponding EAG values were not taken into consideration for statistical analysis. No percentage on bars indicates that all antennae responded. Bars with different letters in the same mating status (in lower case virgin and in capital mated females) represent significant differences among doses (Tukey post hoc test, *p* < 0.05). The asterisk on the pair of columns indicates significant difference between mating status (Student’s *T*-test, *p* < 0.05).

*T. absoluta* females were able to respond to their own pheromone in EAG although, as expected, the elicited EAG response was much lower in females (0.1 mV to 10 µg of TDTA) (Figure 5) than in males (6 mV to 10 µg of TDTA) (Figure 3). Doses of up to 10 µg of both pheromone components were necessary to elicit response on 100% of female antennae (Figure 5A,B), but 10× less (1 µg) of the binary mixture was sufficient to obtain full depolarizations and only 100 ng to attain 75% of responses (Figure 5C). The depolarizations displayed by virgin females when stimulated with 100 µg of the binary mixture were significantly higher than those displayed by mated females (Figure 5C). Detection of the individual pheromone compounds was generally higher in mated females but in no case was the difference significant relative to virgin individuals. Statistical analyses were only conducted on those doses at which all females responded. 

## 4. Discussion

Knowledge of the behavioral and physiological parameters that affect pheromone production and emission has been useful in improving the understanding of the biochemical factors that mediate attractiveness and sexual receptivity by males [54,61,62,63]. In *T. absoluta* there is no great difference in pheromone production by female moths throughout the scotophase and 2 h before and after. However, female age clearly affects pheromone production, with the young females (1–4 days old) producing the highest amount of each pheromone component and the old individuals (11–15 days old) producing the lowest. The optimum pheromone production is maintained at least during the first 10 days after emergence to rapidly decline afterwards. The effect of age on pheromone production has been noticed in a variety of insects. In the corn stalk borer *Sesamia nonagrioides* (Lefèbvre) (Lepidoptera: Noctuidae), the maximum pheromone titre was present in the gland during the 1st and 2nd scotophase after emergence to sharply decrease on the 3rd and 4th day of age [61]. In contrast, females of the western tarnished plant bug *Lygus hesperus* Knight (Heteroptera: Miridae) did not mate until at least 5 days post-emergence, although many produced choriogenic oocytes by the 4th day, and males preferred to mate with females older than 5 days old rather than with younger females [64]. Our results suggest that females rapidly reach the sexual maturity required to ensure rapid and successful matings with conspecific males [40,41,54].

Interactions between insect pheromones and semiochemicals from the host have been long recognized as a key communication system within species [65]. Some insects sequester or acquire host plant compounds and use them as sex pheromones or sex pheromone precursors, while others produce or release sex pheromones in response to specific host plant cues [65,66]. In this way, females could ensure a suitable food source for the progeny [67]. Occasionally, the activity of the pheromone required the concomitant presence of a specific host plant volatile [68]. Tomato leaf odor elicited upwind orientation flight and landing by mated *T. absoluta* females in wind tunnel experiments as well as egg-laying in oviposition choice tests [69]. Also, in electroantennographic assays, female antenna responded to the herbivore-induced plant volatiles hexanal, nonanal, (*Z*)-3-hexenol, methyl salicylate and indole [70]. However, in spite of the discovery of sensory sensilla present in the antennae of *T. absoluta* [71], no interaction between the host plant and production of the pheromone components by virgin or mated females have been described previously to this work. We have noted that both volatile and tactile cues from the host affect pheromone production, especially on virgin females with the highest amount of pheromone being detected upon reception of olfactory and contact signals from the plant. In mated females, pheromone production appears to be also modulated by both types of stimuli, although to a lesser extent. In addition, we have found that virgin or mated male antennae responded to the pheromone components in a dose-dependent manner following a sigmoid curve from 100 ng to 100 µg doses for a maximum response (plateau effect) to TDTA and the pheromone mixture at 10 µg. The mixture elicited slightly higher but non-significant responses than the major component but this does not imply that the minor compound plays no role in the pheromone mixture. As shown in many cases, the minor compounds usually synergize/modulate the response of the major pheromone component to avoid interspecific communication and matings with other sympatric species. Regarding the possible role of contact signals as oviposition cues, we have noticed that antennectomized gravid females are able to oviposit in presence of the tomato plant, with the number of laid eggs being equal to that of naïve females after three days of exposure (unpublished). In contrast, in the absence of the host no oviposition was observed. Thus, it may be possible that structural and/or chemical properties of the plant tissue may trigger oviposition [72,73]. 

A sensitization effect was noticed when male antennae were stimulated at several time intervals after the first exposure to TDTA. The increase of depolarizations from the second puff was apparent in virgin and mated males, the latter individuals being significantly more sensitive than the former. Moreover, while in virgin insects the effect appeared to reach a plateau around 20–30 min after pre-exposure, in mated individuals the effect was still steadily increasing after the last stimulus of the experiment (33 min). It is well known that previous pre-exposure to the pheromone generally results in a reduction of the male attraction to the pheromone [74,75]; however, we and others have described an increased sensitivity to the female sex pheromone of *Spodoptera littoralis* (Boisduval) (Lepidoptera: Noctuidae) by male antennae after a brief exposure to the pheromone components [59,76,77,78]. Male sensitivity to pheromone is a physiological characteristic that varies among moth species and has been linked to the susceptibility of moths to control by pheromone-based mating disruption [79]. Male moths that exhibit a broad dose response to pheromone are often more difficult to disrupt than species with a narrowly defined dose response that are arrested by high pheromone doses [79,80]. This is the first time that the sensitization effect is noted in *T. absoluta,* being particularly remarkable in mated males. This plasticity, related to physiological states and previous experiences, is an adaptative mechanism that allows males to orient more efficiently toward the source of pheromone reducing energy costs. The lifespan of the severed antennae hampers visualization of the real effect along time but no further experiments (behavioral, single sensillum recordings, …) were run to disclose the extension of the effect. 

Autodetection is a term to designate the capability of female insects to detect their own sex pheromone [81]. Initially, it was assumed that pheromones were produced by females and detected by males but this theory soon resulted to be untrue. In an ever-increasing number of cases (see the excellent review on autodetection by Holdcraft et al. [82], the pheromone may induce certain behavioral effects also on females. Thus, pheromone exposure may advance initiation of calling and increase the number of calling females [83,84], or delay the onset of calling and reduce the proportion of calling females and egg-laying [85]. Autodetection of the pheromone may also cause aggregation of females to increase probability of mating success [86] or induce dispersal under high population levels [87]. In our EAG studies, *T. absoluta* virgin females also responded to their own pheromone, particularly to the binary mixture, the response threshold being one-two orders of magnitude lower than the sensitivity displayed by males. Mated females also showed autodetection to the pheromone, the responses to the single pheromone components were similar to those of virgin individuals and lower than the responses to the pheromone mixture. To our knowledge, this is the first time that an insect of the family Gelechiidae shows autodetection to the pheromone [82]. Further investigation is needed to disclose how autodetection of the pheromone affects females’ behavior.

## 5. Conclusions 

Our findings show that *T. absoluta* pheromone production is clearly dependent on female age, the young females producing the highest amount of pheromone, probably to warrant reproduction success. In the presence of the host, virgin and mated females produce higher amounts of TDTA, the major component of the pheromone, than those in the absence of plant and those devoid of olfaction but in physical contact with the plant. In virgin females, pheromone production appears to be stimulated by volatile and tactile cues among other input signals, which may favor host recognition and suitability for offspring success. In electrophysiological assays and for the first time, a sensitization effect to the major component of the pheromone has been noticed, particularly in mated males. Autodetection of their own pheromone by females has also been disclosed for the first time in an insect of the family Gelechiidae. The two last effects should be further investigated to learn the possible implications they may have on the behavior of the pest. Our results will aid to understand the chemical communication of the insect, which may be important for development of improved pheromone-based management strategies for its control.

## Figures and Tables

**Table 1:** Increase of the mean response to 1 µg of TDTA in virgin and mated *T. absoluta* males when stimulated at different times relative to that at T = 0 min.

Status	Time (min)	Increase of Response (mV) ^a^	Percentage of Increase (%)	CI ^b^
Mated	9	0.82	33	(−0.02; 1.67)
	13	1.47 *	58	(0.65; 2.29)
	19	2.55 *	101	(1.72; 3.39)
	24	3.46 *	137	(2.64; 4.28)
	33	4.10 *	163	(3.29; 4.92)
Virgin	9	0.87 *	47	(0.03; 1.70)
	13	1.29 *	70	(0.45; 2.12)
	19	1.67 *	91	(0.80; 2.53)
	24	1.62 *	88	(0.78; 2.45)
	33	1.47 *	80	(0.63; 2.30)

^a^ Asterisks denote statistical significance vs. response at T = 0 at α = 0.05 level. ^b^ 95% Confidence interval.

## Supplementary Materials

The following are available online at https://www.mdpi.com/2075-4450/10/8/256/s1, Figure S1: GLC analysis of a female gland extract and mass spectra of the pheromone components. Figure S2: Pheromone contents of 2-day-old virgin females (N = 5) at 2 h intervals before, during and after the scotophase. Table S1: Amount of pheromone components produced by *T. absoluta* females in the presence/absence of plant. Table S2. EAG response difference of *T. absoluta* males to TDTA at different times vs. at T = 0.
