# Supplementary material for: Influence of Age, Host Plant and Mating Status in Pheromone Production and New Insights on Perception Plasticity in Tuta Absoluta
Source: Insects. 2019 Aug 20;10(8):256. doi: 10.3390/insects10080256 (PMC6722863; doi:10.3390/insects10080256)
Supplement: Supplementary file 1 [file insects-10-00256-s001.zip › insects-528589-SI.pdf]

SUPPLEMENTARY MATERIAL

Influence of age, host plant and mating status in pheromone production and new insights on perception plasticity in *Tuta absoluta*

Aroa Domínguez, Sergio López, Ana Bernabé, Ángel Guerrero* and Carmen Quero*

Department of Biological Chemistry, Institute of Advanced Chemistry of Catalonia (CSIC), Jordi Girona 18. 08034 Barcelona, Spain; aroadc.87@gmail.com (A.D.) slrqbm@cid.csic.es (S.L.); abernamu@gmail.com (A.B.).

* Correspondence: agpqob@cid.csic.es (A.G.); cqlqob@cid.csic.es (C.Q.); Tel. +34-93-4006171.

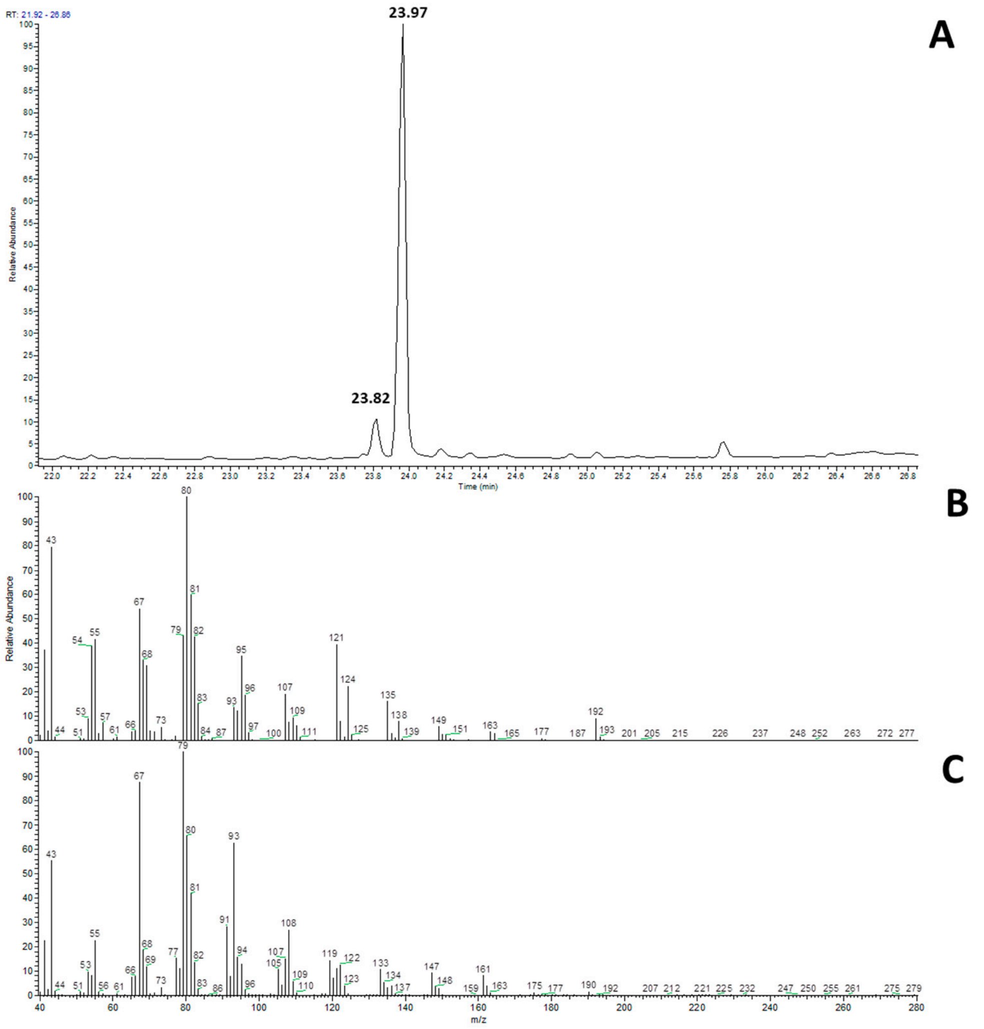

Figure S1. **A**) Partial GLC analysis of a female gland extract of *Tuta absoluta*. **B,C**) Mass spectra of peaks at retention times 23.82 and 23.97 min correspond to the pheromone components (*E*3,*Z*8,*Z*11)-tetradecatrien-1-yl acetate (*E*3,*Z*8,*Z*11-14:Ac) and (*E*3,*Z*8)-tetradecadien-1-yl acetate, respectively

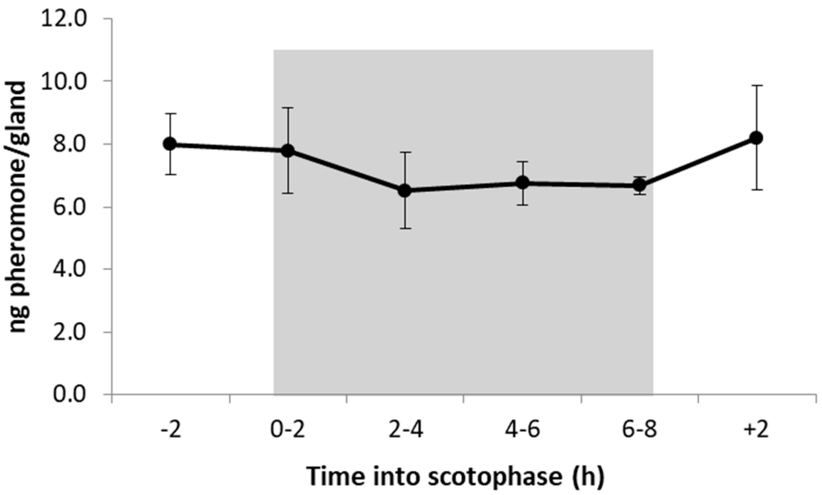

Figure S2. Pheromone contents (± SE) of 2-day-old virgin females (N=5) at 2 h intervals before, during and after the scotophase (shaded grey area).

Table S1. Mean amount (±SD) of the pheromone components TDTA and TDDA detected in gland extracts of virgin and mated *T. absoluta* females[a] in the presence/absence of plant.

**Presence of plant**		**TDTA (ng/gland)**		**TDDA (ng/gland)**	
		**Virgin**	**Mated**	**Virgin**	**Mated**
Without		5.5 ± 0.5D	12.5 ± 1C	0.6 ± 0.1 c	0.9 ± 0.1bc
With	Only olfaction	12.9 ± 1.1BC	12.7 ± 1.9BC	1.0 ± 0.3bc	1.1 ± 0.1bc
	Only contact	12.6 ± 1.5BC	11.4 ± 0.8C	1.0 ± 0.2 bc	0.9 ± 0.1bc
	Olfaction/ contact	26.3 ± 1.8A	17.1 ± 1.7B	2.6 ± 0.1 a	1.2 ± 0.1b

[a]Values within the same mating status followed by different letters are significantly different (P<0.05, Kolmogorov-Smirnov test for TDTA, Tukey´s test for TDDA).

Table S2. Mean EAG response difference of virgin and mated *T. absoluta* males to 1 µg of TDTA at different times *vs* at T = 0[a].

Time (min)	Response difference (mV)	CI[b]
9 *vs* 0	-0.037	(-1.22 ; 1.15)
13 *vs* 0	0.19	(-0.98 ; 1.36)
19 *vs* 0	0.89	(-0.31 ; 2.09)
24 *vs* 0	1.85*	(0.68 ; 3.019)
33 *vs* 0	2.64*	(1.47 ; 3.81)

[a]Asterisks denote statistical significance at $\alpha = 0.05$ level.
[b]95% Confidence interval.
